# Persistence of commensal multidrug-resistant *Escherichia coli* in the broiler production pyramid is best explained by strain recirculation from the rearing environment

**DOI:** 10.3389/fmicb.2024.1406854

**Published:** 2024-07-05

**Authors:** Sébastien Olivier Leclercq, Philippe Bochereau, Isabelle Foubert, Yannick Baumard, Angélique Travel, Benoît Doublet, Sylvie Baucheron

**Affiliations:** ^1^INRAE, Université de Tours, ISP, Nouzilly, France; ^2^INRAE, PEAT, Nouzilly, France; ^3^ITAVI, French Poultry Institute, Nouzilly, France

**Keywords:** *Escherichia coli*, antimicrobial resistance, broiler production pyramid, longitudinal study, whole genome sequencing

## Abstract

Despite the success of mitigation policies in several countries to reduce the use of antibiotics in veterinary medicine, pathogenic and commensal bacteria resistant to antibiotics are still circulating in livestock animals. However, factors contributing the most to antimicrobial resistance (AMR) persistence in these settings are yet not clearly identified. The broiler production, with its highly segmented, pyramidal structure offers an ideal context to understand and control the spread of resistant bacteria. By taking advantage of an experimental facility reproducing the whole broiler production pyramid, we demonstrate that resistant *E. coli* persist in our system primarily though recirculation of a few commensal clones surviving in the rearing environment. No vertical transmission from hens to offspring nor strain acquisition at the hatchery were detected, while import of new strains from outside the facility seems limited. Moreover, each clone carries its own resistance-conferring plasmid(s), and a single putative plasmid horizontal transfer could have been inferred. These results, observed for now in a small experimental facility with high level of biosecurity, must be confirmed in a commercial farm context but still provide invaluable information for future mitigation policies.

## 1 Introduction

Bacterial antimicrobial resistance (AMR) is one of the most important global health threats affecting people at any stage of life, as well as the healthcare, veterinary, and agriculture industries (Antimicrobial Resistance Collaborators, 2022). The wide use of antibiotics in humans and livestock animals has contributed to the selection of resistant bacteria and the spread of AMR worldwide (McEwen and Collignon, 2018). To adapt to antibiotic selection pressure, bacteria have accumulated antibiotic resistance genes (ARGs), leading to multidrug resistant (MDR) phenotypes. Therefore, such ARG accumulation in bacterial pathogens can lead to therapeutic failure (Antimicrobial Resistance Collaborators, 2022). Conjugative elements, such as plasmids, are recognized as a major driver for the spread of ARGs. ARGs can be exchanged between these horizontally-mobile elements by genetic recombination involving insertion sequences, integrons or transposons. Successful plasmids were described to carry numerous ARGs conferring MDR phenotypes including resistances to last generation antibiotics medically-important for public health (e.g., extended-spectrum cephalosporins or carbapenems; Carattoli, 2013; Holmes et al., 2016; Madec and Haenni, 2018; McEwen and Collignon, 2018; Poirel et al., 2018; Leekitcharoenphon et al., 2021). The epidemic success of such MDR plasmids depends both on various co-selections due to antibiotic usage and their spread into and adaptability to different bacterial hosts with which they co-evolve (Madec and Haenni, 2018). Thus, AMR monitoring programs are essential to have comprehensive and reliable information on the emergence and spread of resistant bacteria. They mainly focus on AMR profiles of clinical isolates reported in human and veterinary medicine, but start to routinely screen host-associated commensal bacteria, which may act as ARG reservoir (Madec and Haenni, 2018; Poirel et al., 2018; Racewicz et al., 2022). For instance within the EU, monitoring of AMR in zoonotic and indicator bacteria includes commensal *Escherichia coli* (*E. coli*) from livestock animals and food providing data as an overview, by country and breeding sector (EFSA and ECDC, 2022). *E. coli* is highly-diverse, being commensal as well as pathogenic, and commonly harbors multiple MDR plasmids, hence making it a useful AMR indicator organism (Poirel et al., 2018; Anjum et al., 2021; Leekitcharoenphon et al., 2021). Despite decreasing since few years, MDR *E. coli* isolates in UE are still higher in healthy broilers compared to other livestock animals (38.7% in broilers, 34.2% in pigs and 26.8% in calves in 2018/2019 (EFSA and ECDC, 2022). Particularly, poultry have been shown to play a major role in epidemiology of extend-spectrum β-lactamase (ESBL) and AmpC-type β-lactamase genes, i.e., *bla*_CTX-M-1_ and *bla*_CMY-2_, respectively (Baron et al., 2018; Madec and Haenni, 2018; Poirel et al., 2018; Apostolakos et al., 2019; Dame-Korevaar et al., 2019; Paivarinta et al., 2020; Mikhayel et al., 2021; Mo et al., 2021).

A recent comprehensive review identified four major transmission routes potentially involved in the dissemination of ESBL/AmpC-producing *E. coli* in the broiler production pyramid: (1) breeders-to-broilers vertical transmission, (2) transmission at hatcheries, (3) horizontal transmission between adjacent or successive flocks due to farm-level strain persistence, and (4) between-farm transmission through external factors (Dame-Korevaar et al., 2019). However, the relative contribution of each of these routes to the global AMR dissemination process could not be determined because of a lack of quantitative results and/or a lack of precision in strains genetic relationships. Moreover, the dissemination of resistance plasmids in addition to resistant strains was not considered. More recent studies included high-resolution typing of resistant isolates using whole-genome analyses, and showed that vertical transmission (Apostolakos et al., 2020), persistence of resistant *E. coli* clones and plasmids in the broiler farms (Paivarinta et al., 2020; Mo et al., 2021), as well as between-farm transmissions are possible factors for the AMR burden in the broiler production pyramid. But again, the relative importance of these transmission routes could not be inferred because quantitative comparisons between the different production steps were missing.

Therefore, the aim of the present study was to quantitatively investigate the dynamics of multi-drug resistant commensal *E. coli* spread in the pyramidal broiler production using whole genome sequencing. AMR transmission without antibiotic pressure was followed over 3 generations of broiler and parental chickens in an experimental facility integrating all steps of the broiler production chain. Four main hypotheses were investigated: (i) familial inheritance (i.e., from hens to offspring), (ii) strain acquisition at the hatchery, (iii) recirculation of resistant strains persisting in the rearing environment, and (iv) strain acquisition from outside the facility (i.e., different for each flock or each generation). Several MDR *E. coli* clonal STs were shown to co-exist and persist between chicken generations raised in the same buildings, with no evidence of vertical transmission. Most of these clonal STs were also detected in the environment after disinfection but not at the hatchery, strongly suggesting that the rearing environment is the primary source of flock colonization in the facility. Most resistance genes were shared between STs, however complete plasmid reconstruction indicated that each clonal ST carried its own MDR plasmid, with only one indication of plasmid transfer event detected between different STs.

## 2 Materials and methods

### 2.1 Study design

Resistant *E. coli* strains were monitored from February 2018 to January 2020 in healthy animals and their environments in a French experimental facility integrating all steps of the broiler production chain. The experimental facility is registered for animal experimentation under license number C-37-175-1 of the French Ministry of Agriculture.^1^ We followed the chicken line pHu+ designed to study the meat quality on 6 weeks old broilers (Erensoy et al., 2022). The first generation (G1) of the present study corresponded to the 11th of the breeding history and represented 80 pedigree broiler breeder females. Eleven families of chickens were followed over three generations divided into two sibling batches, one for reproduction which are reared in protected houses during 42 weeks and one for broiler production reared in standard conditions during 6 weeks (Supplementary Tables S1, S2; Supplementary Figure S1).

### 2.2 Sample collection and processing

Individual fecal samples were collected at different weeks of age (1, 3, 6, 20, 32, 42) following standardized protocols specific to age and rearing condition (Baumard et al., 2021). Eggs layed by hens from the followed families were collected at the hatchery during incubation (2 weeks before hatching) of the 2nd and 3rd generations of parental and broiler batches. Additionally, pooled egg shells were collected for each parental hen right after hatching. Environmental samples were collected at the rearing buildings before animal entry using sterile wipes and boot-socks for surfaces, and sterile containers at drinking water supply endpoints. Surfaces and water sprayed to control room moisture at the hatchery were sampled before egg incubation using the same protocols. Each batch of animal feed was also sampled before animal feeding. All samples were immediately stored at 4°C upon return to the microbiology laboratory and processed on the same day.

Fecal and surface samples were inoculated in buffered peptone water (bioMérieux, France) and incubated for enrichment at 37°C for 2 h and for 18 h, respectively. Two-hundred ml of each water sample were filtered on 0.45 μm filter and the filter was directly applied on Mac Conkey agar (bioMérieux, France) plates. After 18 h incubation at 37°C, all bacterial colonies of the filter were inoculated in 20 mL buffered peptone water.

One, two, or three incubated eggs from the same hen were placed into a sterile stomacher filter bag with 33 mL, 66 mL or 200 mL of buffered peptone water, respectively. Eggs were crushed gently for the recovery of bacteria from the shell and those located inside. Hatched egg shells were processed in the same way using 50 mL of buffered peptone water. After 5 min of contact, 10 mL were pipetted from the stomacher filter to a sterile tube and incubated overnight at 37°C for enrichment.

### 2.3 *Escherichia coli* isolation and antibiotic susceptibility testing

Resistant *E. coli* strains were selected on MacConkey agar plates supplemented with ampicillin-tetracycline (50–10 mg/L), ceftriaxone (1 mg/L), enrofloxacin (0.5 mg/L) or sulfonamide-trimethoprim (20–250 mg/L) by plating 100 μL of enrichment cultures. Up to 5 colonies were picked and grown in Luria Bertani broth and stored at - 80°C in 20% glycerol for further analyses. *E. coli* identification was done by specific PCR *uidA* (Heininger et al., 1999) and confirmed for a few isolates by the MALDI Biotyper® system (Bruker Daltonics).

Antibiotic susceptibility was determined by the disks diffusion method on Mueller-Hinton agar (Difco, Sparks, MD), as recommended by EUCAST 2018 guidelines^2^ and CA-SFM 2018^3^ using the following disks (Biorad, Marne-la-coquette, France): Amikacin (AMK, 30 μg), ampicillin (AMP, 30 μg), cephalexin (CXN, 30 μg), chloramphenicol (CHL, 30 μg), ciprofloxacin (CIP, 5 μg), enrofloxacin (ENR, 5 μg), florfenicol (FFC, 30 μg), gentamicin (GEN, 10 μg), kanamycin (KAN, 30 μg), nalidixic acid (NAL, 30 μg), streptomycin (STR, 10 μg), spectinomycin (SPT, 100 μg), sulfonamides (SUL, 200 μg), tetracycline (TET, 30 μg) and trimethoprim (TMP, 5 μg).

Isolates originating from the same animal or surface at the same sampling date and showing the same resistance profile (whatever the initial antibiotic selection) were considered duplicates and only one of them was chosen for further analysis.

### 2.4 Whole-genome sequencing

A set of 178 *E. coli* isolates representative of 5 of the 11 chicken families and of the rearing environment, and including the two main antimicrobial resistance phenotypes (AMP-TET or AMP-SUL-TET-TMP), were subjected to whole-genome sequencing (WGS). Briefly, DNA extraction was performed using the Nucleospin 8 tissue kit (Macherey-Nagel, Düren, Germany). DNA samples were sent to Novogene Co., Ltd., for library preparation using the New England Bioloabs Next Ultra DNA library prep kit and WGS on NovaSeq (Illumina) using 2 × 150-bp paired-end sequencing (Novogene, Bejing, China).

A subset of 42 isolates, including at least two isolates for each major *E. coli* sequence type (ST) observed in this study and one for other ST, was subjected to WGS using the Oxford Nanopore technologies (Oxford, United Kingdom). Briefly, libraries preparations were done with the Rapid barcoding sequence kit (SQK-RBK 004) and MinION sequencing runs were performed on standard flow cells (FLO-MIN106).

### 2.5 *In silico* analyses

Raw Illumina reads quality was assessed using FastQC^4^; all reads had >20 phred scores at every base, suitable for assembly. Residual sequencing adapters were removed using trimmomatic 0.39 (Bolger et al., 2014). Genomes were assembled using SPAdes 3.15.3 (Bankevich et al., 2012) with the best kmer selected among 21, 33 or 55, the careful correction option set to “ON” and no coverage cutoff. Assemblies were then filtered to remove contigs shorter than 200 bp and with a coverage ratio smaller than 0.2 compared to scaffolds larger than 50,000 bp. Sequence types (ST), antibiotic resistance genes, chromosomal mutations conferring resistance, and plasmid replicon markers were detected for each assembled genome using the starAMR tool v.0.8 (Bharat et al., 2022) with mlst, Resfinder, Pointfinder, and Plasmidfinder databases version 2.19.0, 05/24/2022, 02/01/2021 and 11/29/2021, respectively. Parameters for starAMR were set to ‘ecoli’ for the mlst scheme, ‘escherichia_coli’ for the pointfinder organism, and 60 and 95% for minimum coverage and identity hit reporting thresholds, respectively. Phylogroups were predicted using ClermontTyping (Beghain et al., 2018) v.1.4.1 with the Mash method, and serotypes were predicted using SerotypeFinder (Joensen et al., 2015) with the same thresholds as above. Maximum-likelihood (ML) phylogenetic trees covering the different isolates were obtained by analysis of the core-genome SNPs using ParSNP v.1.2 (Treangen et al., 2014), with the assembled genomes as input, the MUMi filtering disabled (option -c) and the *E. fergusonii* RHB38-7 genome (accession CP057093) as outgroup. SNP distances between isolates belonging to the same ST were calculated with snp-dists^5^ on core-genome SNPs returned by ParSNP (covering 65–76% of the genomes), using default parameters.

MinION sequencing reads with an average quality higher or equal to 7 (phred score) and a minimum size of 500 bp were selected with nanofilt v.2.5.0 and given as input, along with corresponding clean Illumina reads, to Unicycler v.0.4.7 (Wick et al., 2017) with default settings to perform a hybrid assembly. Due to a large amount of raw reads, the quality filter was set to 10 for samples F60, F163, F719, F730, and F1055. Assembled scaffold were then annotated using DFAST v.1.2.14 (Tanizawa et al., 2018) with HMM search on TIGRFAM and CDD enabled and a contig size filter of 200 bp. In addition, Resfinder v4.1, ISfinder, and a home-made plasmid conjugation protein database were used as custom databases with, respectively, 100, 98 and 90% similarity thresholds to increase annotation sensitivity for genes related to antibiotic resistance dissemination. Plasmid sequence types of IncF replicons were identified using the pMLST tool v0.1.0 with standard parameters and database version 2023-04-24 (Carattoli et al., 2014). The IncF plasmid phylogenetic analysis was performed as follows: a ~ 24 Kbp region of IncF plasmids containing genes encoding the conjugative machinery (*tra* genes) was extracted from one representative isolate of each ST and searched against the 34,513 complete plasmids included in the PLSDB database version 2021 (Schmartz et al., 2022) using blastN. The two best matches were kept for each input plasmid and a global alignment of the *tra* region was performed using MAFFT v7.490 (Katoh and Standley, 2013) with default parameters. A maximum likelihood phylogenetic tree was computed from the alignment using IQ-TREE v2.2.0 (Nguyen et al., 2015) with 1,000 bootstrap replicates. All phylogenetic trees were displayed and annotated using iTOL v6 (Letunic and Bork, 2021). Plasmid comparison figures were produced with Genofig v1.1.^6^ (Branger and Leclercq, 2024).

### 2.6 Statistical analyses

The impact of animal family, age, generation and rearing condition on isolates ST was investigated using *χ*^2^ statistics. The 104 tetra-resistant and 33 bi-resistant sequenced isolates originating, respectively, from the 6 and 3 major clonal ST were selected. Only isolates originating from feces were considered. *χ*^2^ tests were performed between each pair of variables (ST, family, age, generation, rearing condition) using R v4.2.2. A Bonferroni correction for multiple tests was applied by multiplying the resulting *p-values* by the number of tests performed (ten). Correlations between the ST and the other variables were considered significant for corrected *p-value* lower or equal to 0.05. The same procedure was applied for tetra-resistant clonal ST isolates associated only to parental animals or broilers with a Bonferroni correction set to six.

## 3 Results

### 3.1 Description of the sampling campaign in the facility

Animals were followed for three generations (G1, G2, and G3) in two rearing conditions (Parental and Broiler), and sampling was restricted to 11 hens at G1 and their descendants at G2 and G3 (Supplementary Figure S1). A total of 335 fecal samples were collected from 82 animals evenly distributed among the selected families and among generation, rearing condition, and age (Table 1; Supplementary Figure S1). Sampling started at 20 weeks of age for the first generation, and could not include broiler animals which are slaughtered after 6 weeks in the studied breed (Supplementary Figure S1). Twenty-eight environmental samples were also collected from building surfaces and drinking water the day before animal’s entry (Table 1). A total of 613 non-duplicate resistant *E. coli* strains were isolated from these samples using either AMP- TET, ENR or SUL- TMP agar plates (Table 1). Animal feed, surfaces and water at the hatchery, as well as incubated eggs and post-hachting eggshells were also sampled but no AMR *E. coli* could be isolated (Table 1).

**Table 1:** Number of samples and corresponding resistant *E. coli* isolates.

		Parental hens (samples/isolates)									Broiler group (samples/isolates)					
		w-2	d-1	w1	w3	w6	w20	w30	w42	Total	w-2	d-1	w1	w3	w6	Total
Feces	G1	–	–	–	–	–	11/10	11/10	11/4	**33/24**	–	–	–	–	–	–
	G2	–	–	15/12	15/20	15/52	14/31	13/29	13/19	**85/163**	–	–	20/14	22/23	23/66	**65/103**
	G3	–	–	18/12	17/21	18/51	14/24	14/19	14/25	**95/152**	–	–	19/40	19/47	19/64	**57/151**
Eggs	G2	16/0	–	–	–	–	–	–	–	**16/0**	5/0	–	–	–	–	**5/0**
	G3	27/0	–	–	–	–	–	–	–	**27/0**	33/0	–	–	–	–	**33/0**
Hatcherysurfaces	G2	4/0	–	–	–	–	–	–	–	**4/0**	4/0	–	–	–	–	**4/0**	G3	3/0	–	–	–	–	–	–	–	**3/0**	8/0	–	–	–	–	**8/0**
Rearing building surfaces	G2	–	7/8	–	–	–	–	–	–	**7/8**	–	4/0	–	–	–	**4/0**
	G3	–	5/7	–	–	–	–	–	–	**5/7**	–	4/4	–	–	–	**4/4**
Drinking water	G2	–	3/1	–	–	–	–	–	–	**3/1**	–	0/0	–	–	–	**0/0**
	G3	–	3/0	–	–	–	–	–	–	**3/0**	–	2/0	–	–	–	**2/0**
Food	G1	–	–	–	–	–	2/0	2/0	–	**4/0**	–	–	–	–	–	
	G2	–	1/0	–	–	1/0	2/0	2/0	–	**6/0**	–	1/0	1/0	–	–	**2/0**
	G3	–	1/0	–	1/0	–	2/0	1/0	–	**5/0**	–	1/0	–	1/0	–	**2/0**

w, week; d, day; G, generation of chickens; −: no sample collected.

### 3.2 Antibiotic resistance profile of fecal *Escherichia coli* isolates

The 593 *E. coli* isolated from feces were resistant to 4 antibiotics on average, from the set of 15 screened antibiotics. Most isolates were resistant to AMP (88%) and TET (78%), while more than half were resistant to SUL (60%), STR (57%) or TMP (55%). A substantial proportion of isolates were also resistant to the fluoroquinolone CIP (24%) and to a lesser extent to ENR (15%), mainly due to differences in resistance breakpoint definitions between human antibiotics (EUCAST 2018 guidelines) and veterinary antibiotics (CA-SFM 2018 guidelines). Resistances to CHL, FFC or KAN were also observed, but at a much lower rate (<5%). Notably, no resistance was observed to third generation cephalosporins (ceftriaxone, CRO). The average number of resistance per isolate ranged from 3.6 to 4.6 between chicken families but the difference was not statistically significant (Supplementary Figure S2A, Kruskall-Wallis H test, *p-value* = 0.306). At the flock level, strains isolated from the same rearing condition had a similar average number of resistance over the second and third generations (~3.6–4 for parental and 4.5–4.9 for broiler groups), while broiler isolates were statistically more resistant than parental isolates (all Wilcoxon corrected *p*-values <0.05). The level of resistance of parental isolates from the first generation was unexpectedly low compared to the two other generations, with only 2.5 resistance per isolate on average, which led to highly significant differences with all the other flocks (all Wilcoxon corrected *p*-values <10^−3^).

**Figure 1:**
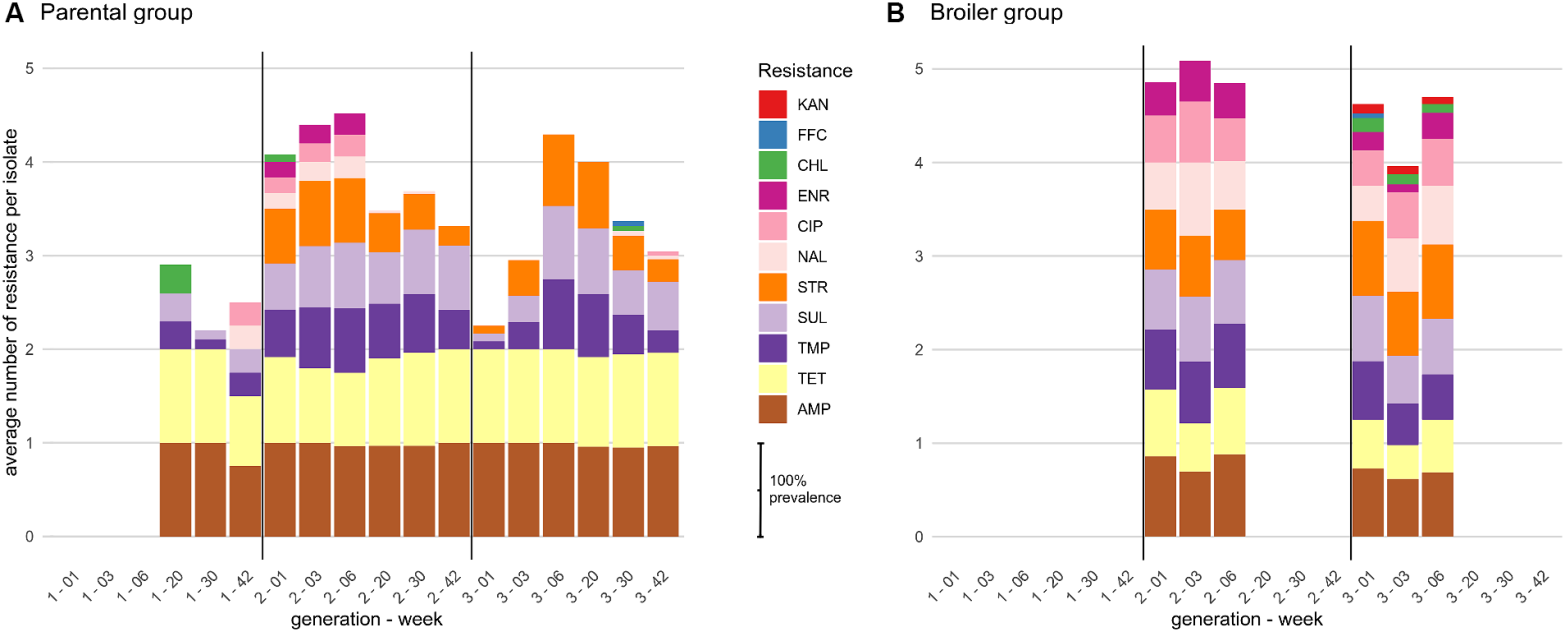
Distribution of phenotypic resistances among *E. coli* isolates for **(A)** parental and **(B)** broiler chicken. Bars represent the cumulative prevalence of isolates at each week of age resistant to at least two antibiotics, calculated antibiotic by antibiotic. Broilers are slaughtered at 6 weeks of age. Sampling started at week 20 for generation 1. AMP, ampicillin; TET, tetracycline; TMP, trimethoprim; SUL, sulfonamides; STR, streptomycin; NAL, nalidixic acid; CIP, ciprofloxacin; ENR, enrofloxacin; CHL, chloramphenicol; FFC, florfenicol; KAN, kanamycin.

The difference in average resistance between parental and broiler groups was mainly driven by the resistance to (fluoro-)quinolones. Indeed, more than 50% of isolates were resistant to CIP and/or NAL at every sampling date and generation of broiler animals while in the parental group, isolates with these resistances were found almost only in young animals (6 weeks or less) of the second generation (Figure 1). Isolates resistant to CHL or KAN were detected in all sampling dates of the broiler G3 condition and only sporadically in other flocks, but their low prevalence (max 15% of the isolates at the first week of age) did not impact the average number of resistances per isolate compared to the broiler G2 group. Interestingly, the lower average number of resistance in parental G1 isolates is well explained by the total lack of STR resistance otherwise found in all other flocks, and by the lower proportion of isolates resistant to SUL and/or TMP (Figure 1). Apart from the small between-flock differences described above, observed resistances were highly similar between generation and rearing condition, with 77% of all isolates resistant to at least AMP-TET (49–98%, depending on the condition) and 43% to at least AMP-SUL-TET-TMP (21–49% depending on the condition). Finally, no family-specific pattern of resistance was observed (Supplementary Figure S2B).

### 3.3 Phylogenetic relationship between representative MDR isolates

The widespread prevalence of the tetra-resistance phenotype AMP-SUL-TET-TMP in *E. coli* isolated during this study may indicate the persistence of a dominant MDR clone in the facility, or the efficient dissemination of a resistance plasmid among the facility’s *E. coli* population. To investigate these two hypotheses, a subset of 113 fecal isolates showing resistance to at least four antibiotics, representative of the chicken generations and rearing conditions from 5 chicken families, were subjected to a whole genome sequence analysis (Supplementary Table S3). Fourteen isolates from rearing environment surfaces sampled before flock entry and showing the aforementioned tetra-resistance were also included to investigate a potential environmental persistence.

**Figure 2:**
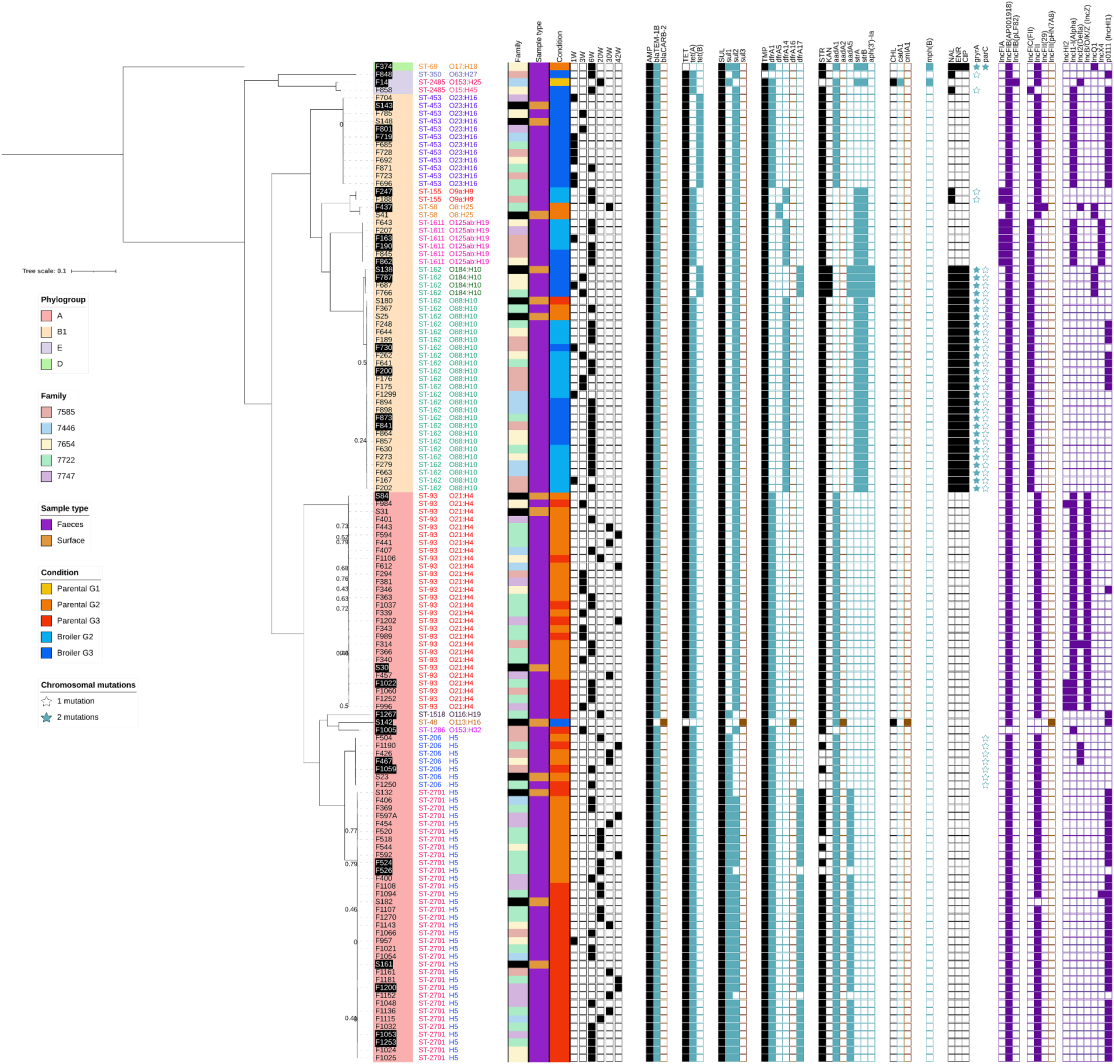
Phylogenetic relationship of 127 tetra-resistant *E. coli* strains originating from feces and rearing surfaces before animal’s entry. The tree was constructed with a maximum likelihood algorithm from the core genome SNP alignment. *E. fergusonii* was used as an outgroup but is not shown on the figure to improve branch length visibility. Branch confidence values of 0.8 or higher are not displayed. For each antibiotic, the resistance phenotype is symbolized with a black filled square while presence of associated resistance gene(s) is indicated with blue or brown filled squares. Brown squares represent genes detected in surface isolates only. Chromosomal mutations leading to fluoroquinolone resistance are indicated with open or filled stars. Samples highlighted in black were assembled using both short and long reads.

Isolates were distributed among four *E. coli* phylogroups (A, B1, D, and E) with large over-representation of phylogroup A (57%) and B1 (40%). A total of 14 sequence types (ST) was observed but 91.4% of the sequenced isolates clustered in only 6 major ST: ST-453, ST-1611, ST-162, ST-93, ST-206 and ST-2701 (Figure 2). Each of them was represented by a single clonal group (<25 SNPs between each pair of isolates) with two exceptions: (1) ST-162 was separated into two clonal groups differentiated by their inferred serotypes (O184:H10 and O88:H10) and separated by 1866–1875 SNPs, and (2) ST-93 showed a slightly higher diversity (0–188 SNPs, average 38) with two heterogeneous subgroups separated by only 20–50 SNPs. ST-93 can therefore be considered as a single clonal group with a higher mutation rate or originating from an older common ancestor (Duval et al., 2023). The distribution of these seven major clonal groups was highly correlated with the rearing condition (*χ*^2^ test corrected *p-value*: 2.5×10^−18^): all fecal isolates of the three ST from phylogroup A (ST-93, ST-206 and ST-2701) originated from parental animals, while those of the four ST from phylogroup B1 (ST-453, ST-1611, ST-162_O184:H10, ST-162_O88:H10) originated at 98% (41/42 isolates) from broiler animals. No effect of the family could be detected on the clonal group distribution among animals (*χ*^2^ test corrected *p-value*: 0.81) while a significant effect of animal age and generation was observed (*χ*^2^ test corrected *p-values*: 5.9×10^−8^ and 8.7×10^−3^, respectively). Impacts of age and generation disappeared when only parental-associated isolates were considered (*χ*^2^ test corrected *p-values*: 0.053 and 0.45, respectively), but were still significant for broiled-associated isolates (*χ*^2^ test corrected *p-values*: 0.01 and 0.005, respectively) probably caused by ST-453 isolates exclusively observed in G3 animals and mostly at week 1 (Figure 2). Interestingly, isolates from all these clonal groups but one (ST-1611) were also detected on surfaces before entry of animals, in the same rearing conditions than their fecal counterparts.

The same analysis was performed on a representative set of AMP-TET resistant isolates originating from feces (*n* = 47), surfaces (*n* = 3) and drinking water (*n* = 1) in order to evaluate if the major MDR ST described above evolved from less resistant clones already circulating in the facility. These bi-resistant isolates also clustered in a few clonal ST (inter-isolate differences <20 SNPs), whose distribution were again correlated with the rearing condition (*χ*^2^ test corrected *p-value*: 6.8×10^−7^) but not to any other parameter (Supplementary Figure S3). None of these clonal ST matched to any of the six major tetra-resistant STs, indicating that bi- and tetra-resistant prevalent strains belonged to independent *E. coli* populations.

Overall, the extremely low between-isolates genomic distance within each major ST and their strong association with the rearing condition over generations indicate that the primary factor explaining the persistence of resistant *E. coli* in our facility is a horizontal transmission of a few MDR clones between successive flocks, likely through recirculation from the rearing environment.

### 3.4 Antibiotic resistance gene content in selected isolates

The ARG content of sequenced isolates was then investigated to characterize the genetic determinants leading to their resistance phenotype. Twenty-five acquired ARGs were detected in total (Figure 2; Supplementary Figure S3), and a very good genotype-to-phenotype match was obtained, except for some ST-206 and ST-2701 isolates sensitive to streptomycin despite the carriage of *aadA1* and/or *aadA5*. *bla*_TEM-1B_, *sul2*, and *tet*(A) were the most widely disseminated genes, respectively found in all, six, and five of the seven tetra-resistant clonal groups. The gene *dfrA1* was the most disseminated TMP resistance determinant, being found in four of the seven clonal groups. Streptomycin resistance was conferred either by *aadA*1/*aadA*5 or by *strAB* in the major STs, while the presence of *aph*(3′)-Ia correlated well with the extended aminoglycoside resistance to kanamycin of the ST-162_O184:H10 clonal group (Figure 2). Seven ARGs were detected only in one or two isolates (*bla*_CARB-2_, *sul3*, *dfrA16*, *aadA2*, *catA1*, *cmlA5*, *mph(B)*), while the two β-lactamase gene variants *bla*_TEM-1A_ and *bla*_TEM-1D_ were found only in AMP-TET resistant bacteria (Figure 2; Supplementary Figure S3). Finally, quinolone phenotypic resistance was essentially restricted to ST-162 isolates and a single phylogroup E isolate. ENR/CIP-resistant strains showed typical D87N/D87Y and S83L mutations in *gyrA* and S80I mutation in *parC*, which are the most common (fluoro)-quinolone resistance mechanism in *E. coli* (Fabrega et al., 2009). No plasmid-mediated quinolone resistance genes were detected.

In summary, all isolates from the well-disseminated tetra-resistant clonal groups harbor a similar ARG repertoire, despite large phylogenetic distances between these clones. This situation suggests that the ubiquity of the tetra-resistance profile may be caused by the dissemination of one or two major MDR plasmids or other conjugative elements in the facility.

### 3.5 Antibiotic resistance gene-carrying plasmids diversity in the various *Escherichia coli* clonal groups

Analysis of plasmid replicon markers detected in our 178 draft assembled genomes suggested the presence of an IncF replicon in 100% of tetra-resistant isolates and in 90% of AMP-TET isolates (Figure 2). IncHI1 and IncI1 replicons were the second and third most abundant replicon types, detected in three of the seven major clonal groups and in several AMP-TET isolates. Seven other replicon types were more sporadically detected although IncZ and IncX4 types were each found in all isolates of one of the major STs. However, no clear ARG-plasmid association could be inferred from Illumina-based assemblies.

Oxford Nanopore sequencing of a subset of 31 tetra-resistant and 11 AMP-TET resistant isolates (black-boxed in Figure 2; Supplementary Figure S3) was therefore performed to link each ARG to its genetic context among the various STs. Within a given clonal group, ARGs were usually carried by almost identical replicons in all isolates (Supplementary Figures S4–S9). Contrary to the high intra-clonal ST homogeneity, ARG-carrying plasmids were much more diverse between clonal STs. All ARGs were located on IncF plasmids in ST-1611 and ST-162 isolates, while most ARGs were located on IncI1 or IncZ plasmids in ST-453 and ST-93, respectively (Table 2). In ST-2701, ARGs were distributed between IncF and IncHI1-related plasmids. Most ARGs were found on different plasmid types, with the exception of *tet*(A) detected only on IncF plasmids in the six major STs. However, *tet*(A) was also carried by IncI1, IncN and IncH1 plasmids in minor STs and AMP-TET resistant isolates (Supplementary Tables S4, S5), ruling out a specific IncF-related dissemination of this gene in the facility. A number of ARGs were also detected at chromosomal positions (Table 2; Supplementary Table S4). For instance, *tet*(B) in ST-453 isolates was part of the Tn10 transposon (Chalmers et al., 2000), within a 60 Kbp region precisely integrated in a threonine tRNA (Supplementary Figure S3), while *sul2* in ST-1611 isolates was part of a truncated CR2-*sul2* unit (Zhang et al., 2022) itself located in a larger mobile region integrated in a phenylalanine tRNA (Supplementary Figure S5). These regions had no large-scale homology to any integrative element reported in the ICEberg database (Liu et al., 2019), but harbored some hallmarks of IMEs (Integrative and Mobilisable Elements) such as genes putatively involved in integration and conjugative transfer.

**Table 2:** Genetic context of ARGs detected in the seven major tetra-resistant clonal groups.

Origin	ST	Chr.	IncF	IncF ST	IncI1	IncZ	IncHI1
Broiler	ST-453	*tet*(B)	Ø	F24:A-:B58	*bla*_TEM-1B_ *, dfrA1, aadA1, sul2*	–	Ø
Broiler	ST-1611	*sul2*	*tet*(A)*, bla*_TEM-1B_*, dfrA14, strA, strB, sul2*	C4:A-:B58	Ø	–	–
Broiler	ST-162 (O184:H10)	–	*tet*(B)*, bla*_TEM-1B_*, dfrA17, aadA5, aph(3′)-Ia, strA, strB, sul2*	C4:A-:B58	–	–	–
Broiler and Parental	ST-162 (O88:H10)	–	*tet*(A)*, bla*_TEM-1B_*, dfrA14, strA, strB, sul2*	C4:A-:B58	–	–	Ø^a^
Parental	ST-93	–	*tet*(A)	F24:A-:B58	Ø	*bla*_TEM-1B_ *, dfrA1, aadA1, sul2*	–
Parental	ST-206	–	*tet*(A)*, bla*_TEM-1B_*, dfrA1, aadA1, sul1*	F24:A-:B6	–	–	–
Parental	ST-2701	–	*tet*(A)*, bla*_TEM-1B_*, dfrA1, aadA1, sul1*^b^	F24:A-:B6	–	–	*dfrA17*, *aadA5*, *sul2*^a,b^

Ø, plasmid present but not carrying any known resistance gene. ^a^plasmid not found in all isolates. ^b^plasmid cointegrates in some isolates.

**Figure 3:**
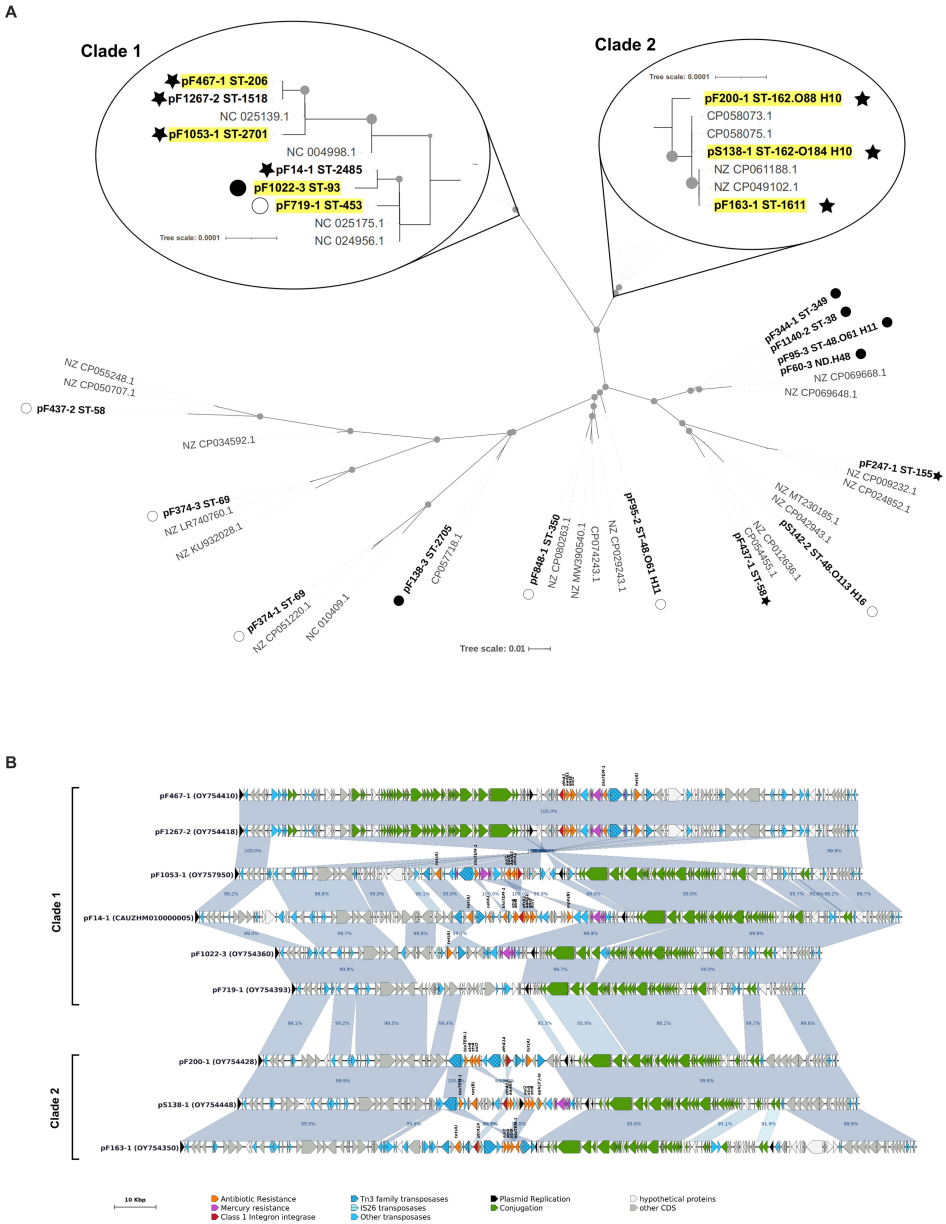
**(A)** IncF plasmids phylogenetic tree. Plasmids from this study are labeled in bold and closest plasmids from the PLSDB database are labeled in gray. Plasmids carried by strains belonging to the six major tetra-resistant ST are outlined in yellow. Only one plasmid by ST is included. Dotted nodes show a bootstrap value >90%. Symbols represent ARG content: star, tetra-resistance determinants; closed circle, AMP and/or TET resistance determinant only; open circle, no ARG detected. **(B)** Graphical representation of IncF plasmids belonging to the two clades zoomed in **(A)**. Antibiotic resistance gene names are not labeled on every sequence to avoid redundancy. Only homologies larger than 2.5 Kbp are displayed.

Since a majority of resistance plasmids were of the IncF incompatibility group, these plasmids were investigated in more details. A plasmid MLST analysis revealed 13 ST among the 22 distinct IncF replicons found in our isolates, indicating a great diversity (Table 2; Supplementary Tables S4, S5). Nonetheless, 6 of the 13 ST showed the B58 allele and could potentially have originated from a unique plasmid which have entered the facility years ago. Publicly available plasmids closest to those of our isolates were therefore collected from the PLSDB complete plasmid database (more than 34,000 replicons at the collection date), and a phylogenetic analysis on the *tra* region was conducted. Most replicons from minor STs and AMP-TET isolates were distantly related to each other, and were intermingled with publicly available plasmids (Figure 3A). IncF plasmids from clonal STs ST-453, ST-93, ST-206, and ST-2701 were extremely closely related (less than 10 mutations over the 25 Kbp aligned region) and formed a well-defined clade with two replicons from minor STs (ST-1518 and ST-2485). They showed a well conserved synteny in their core genome (except a single large inversion in ST-206 and ST-1518 isolates) but their MDR region was highly rearranged (Figure 3B), consistent with the high heterogeneity of ARG content described in Table 2. One exception was the identity between IncF replicons carried by the MDR isolate F1267 (ST-1518) and by ST-206 isolates. Interestingly, ST-1518 isolates from the same serogroup were detected among AMP-TET resistant isolates, but not carrying any IncF plasmid (Supplementary Table S5). We therefore suspect that this IncF replicon may have transferred horizontally between ST-206 and ST-1518 isolates inside the facility, although MDR and AMP-TET isolates did not originate from the same rearing condition or generation (Figure 2; Supplementary Figure S3). Other plasmids from this clade are unlikely the result of an intra-facility dissemination, as suggested by the divergence in the MDR region and the presence of PLSDB plasmids scattered thorough the clade (Figure 3A). IncF plasmids of the two other major MDR STs (ST-1611 and ST-162) showed similar trends, located in a well-defined phylogenetic clade showing a very conserved core region and a heavily rearranged MDR region (Figure 3B). Again, the clade contained plasmids from PLSDB, ruling out a facility-specific dissemination of these plasmids.

## 4 Discussion

It is now widely recognized that the gut of healthy animals is a reservoir of antibiotic resistance genes, carried by commensal bacteria (Poirel et al., 2018). However, how animals acquire resistant gut commensals remains unclear. In the broiler production, chicks are bought at one-day old from hatcheries which get eggs to other facilities raising breeder animals. In this system, chicks and hens are never in contact, and eggs are decontaminated upon introduction in the hatchery (Dierikx et al., 2013). Nevertheless, several studies concluded on the possibility of parent-to-offspring bacterial transmission, either through colonized shell or inner egg (Bortolaia et al., 2010; Agerso et al., 2014; Pedroso and Lee, 2015; Ding et al., 2017; Poulsen et al., 2017). Others also highlighted the hatchery as a potential hub for spreading of fluoroquinolone-resistant *E. coli* or *bla*_CMY-2_-carrying plasmids in different production farms (Petersen et al., 2006; Baron et al., 2018). The experimental facility used in the present study follows the physical separation of hens and chicks as well as egg decontamination in a dedicated hatchery, and we could not see any effect of the chicken family on resistant *E. coli* carriage. More importantly, parental and broiler animals of the same generation harbored completely distinct *E. coli* populations while originating from the same mothers and the same hatchery. Finally, we were not able to isolate any resistant *E. coli* strain from eggs or hatchery samples collected during the experiment. A recent study demonstrated that APEC strains usually reach the oviduct during systemic infection and in some rare occasion infect layed eggs (Abdelhamid et al., 2024), mirroring what has been described for *Salmonella* (Gantois et al., 2009). Although infected eggs usually do not develop properly, it is not known whether some may still be able to hatch, paving the way for a possible transmission to chicks. Such transmission route is less likely to occur for commensal strains lacking the virulence toolbox necessary for systemic infection, which may partly explains our lack of *E. coli* detection in the 81 tested eggs. Nonetheless, it indicates that even if some vertical transmission or contamination at the hatchery could happen, it had a minor effect on the carriage of resistant *E. coli* in this experimental facility.

Several other studies looking at commensal or pathogenic *E. coli* in the broiler production chain also concluded on the lack of vertical transmission, but could not infer the origin of acquired strains (Dierikx et al., 2013; Oikarainen et al., 2019). The most likely route for *E. coli* acquisition in the present study is from the rearing environment. We show evidence that animals from different generations reared in the same building carry the same resistant *E. coli* clones, regardless of the strains carried by their parents. These building-specific clones were also systematically found on the building surfaces before animal’s entry despite between-flock extensive decontamination, indicating an environmental persistence. Dierikx et al. (2013) also reported the persistence of multidrug resistant *E. coli* in the rearing environment of commercial broiler farms after decontamination and the transmission to the next flock, suggesting that transmission through the rearing environment is probably a common phenomenon. This is in line with Rychlik’s observations, showing that *E. coli* is primarily acquired from the environment (Rychlik, 2020). It should be noted that our observations mostly explain farm-level AMR persistence, while rare vertical transmission from breeders or hatcheries, as well as through external factors (transportation equipment, contaminated food, wildlife) should not be neglected as potential factors impacting the spread of AMR between facilities, as proposed for the fast dissemination of APEC O78:H4 strains in parental and broiler farms in Nordic countries (Ronco et al., 2017).

Although no antibiotics was used in the followed breed for the last 10 years, a large amount of multi-resistant *E. coli* was isolated. The genetic determinants of their resistance phenotypes (*bla*_TEM-1_, *tet*(A)/*tet*(B), *sul1*/*sul2*, *aadA1* and *dfrA1*) are extremely common in commensal *E. coli* of production animals (Leekitcharoenphon et al., 2021; Szmolka et al., 2021; Gambi et al., 2022) and were already identified as a “common multi-resistance pattern” in this species (Szmolka and Nagy, 2013). These genes confer resistance to “old” antibiotics widely used in livestock prophylactic treatments or as growth promoters in the last 50 years (Samanidou and Evaggelopoulou, 2008). Their presence in the experimental facility devoid of antibiotic pressure is therefore not surprising and reflect what is observed in commercial organic farms (Gambi et al., 2022).

Even if resistance genes were similar between the various isolated *E. coli*, they were carried by distinct, ST-dependent plasmids. Comparisons with the public plasmid database PLSDB indicated that IncF plasmids found in major clonal STs are all more closely related to plasmids outside the facility than to each other, suggesting that they came along with their current *E. coli* hosts rather than spreading in the *E. coli* population of the facility after a single initial introduction. Nonetheless, most resistance plasmids were able to transfer *in vitro* during conjugation assays and a likely transfer event between a major (ST-206) and a minor (ST-1518) clonal ST was observed, ruling out a lack of dissemination because of plasmid non-functionality.

Plasmid persistence in environments without clear selective pressure, dubbed as the plasmid paradox, have long been observed in the lab as well as in nature but the underlying mechanisms are not fully understood yet (MacLean and San Millan, 2015; San Millan and MacLean, 2017; Carroll and Wong, 2018; Wein et al., 2019). Plasmid-encoded toxin-antitoxin and partition systems are efficient in avoiding plasmid loss over time but do not prevent extinction of the plasmid-carrying population to the benefit of plasmid-free populations with a better fitness. Compensatory mutations in the host and plasmid genomes, which reduce the fitness burden of carrying such plasmids, is another mechanism proposed to play a role in plasmid maintenance (Carroll and Wong, 2018). The multiple rearrangements we observed in the MDR region of IncF plasmids could participate in the reduction of fitness burden, for instance by modulating the regulation of resistance genes. The clear host-plasmid association over chicken generations in the present facility is also in favor of host-encoded compensatory mutations which could have resulted in a co-adaptation of both parties and a reduction of plasmid carriage cost.

In conclusion, our experimental design in which the pedigree of every animal was known and our use of whole-genome sequencing approaches allowed us to quantitatively determine the most likely origin of multi-resistant *E. coli* present within the facility. Vertical inheritance through eggs contamination or acquisition at the hatchery seem inexistent in our study, while we pointed out a very strong role of environmental acquisition of building-specific, persistent strains. We also showed that these persistent strains carried their own resistance plasmids, indicating a limited impact of horizontal gene transfer. These results, although providing a clear explanation on how MDR *E. coli* persist in our facility, were obtained in a well-controlled system and without antibiotic pressure. Applying the same methodology in the more open context of commercial farms will be necessary to know if our results can be generalized to the whole poultry production pyramid.

## Data availability statement

The datasets presented in this study can be found in online repositories. The names of the repository/repositories and accession number(s) can be found in the article/Supplementary material.

## Ethics statement

The animal study was approved by Ministère Français de la Recherche et de l’Enseignement Supérieur (project n° 00880.02). The study was conducted in accordance with the local legislation and institutional requirements.

## Author contributions

SL: Conceptualization, Formal analysis, Investigation, Resources, Software, Validation, Visualization, Writing – original draft, Writing – review & editing. PB: Formal analysis, Investigation, Software, Writing – review & editing. IF: Investigation, Resources, Writing – review & editing. YB: Methodology, Resources, Writing – review & editing. AT: Conceptualization, Funding acquisition, Resources, Writing – review & editing. BD: Conceptualization, Funding acquisition, Investigation, Resources, Validation, Writing – original draft, Writing – review & editing. SB: Conceptualization, Data curation, Funding acquisition, Investigation, Methodology, Project administration, Resources, Supervision, Validation, Writing – original draft, Writing – review & editing.

## Footnotes

^1^https://doi.org/10.15454/1.5572326250887292E12

^2^http://www.eucast.org/

^3^https://www.sfm-microbiologie.org/boutique/comite-de-lantibiograme-de-la-sfm-casfm/

^4^https://github.com/s-andrews/FastQC

^5^https://github.com/tseemann/snp-dists

^6^https://forgemia.inra.fr/public-pgba/genofig
