# Supplementary material for: Persistence of commensal multidrug-resistant Escherichia coli in the broiler production pyramid is best explained by strain recirculation from the rearing environment
Source: Front Microbiol. 2024 Jul 5;15:1406854. doi: 10.3389/fmicb.2024.1406854 (PMC11259971; doi:10.3389/fmicb.2024.1406854)
Supplement: Supplementary file 1 [file Data_Sheet_1.pdf]

Table S1 Rearing protocol

Age		Parental flocks		Broiler flocks
	**Hatching / hatchery**			
		By Pedigree in protected area		By Pedigree in protected area
	**Rearing**			
**w1-6**		On floor (n=540 ; d=5)		On floor (n=450; d=6.4)
**w7-18**		On floor		
**w18-42**		Individual cages (n=248)		
	**Houses**			
		Overpressure and filtered air<br>Limited access to authorized personnel<br>in compliance with biosafety instructions		Standard rearing conditions
	**Temperature**			
**d1**		31°C		31°C
**w1-6**		31-20°C (gradual decrease)		31-20°C (gradual decrease)
**w7-w42**		20°C		
		**Female**	**Male**	
	**Light program** (H)			
**d1-d3**		24	24	24
**d4-d7**		16	16	24
**w2-6**		8	8	18
**w7-17**		8	8	
**w18**		9	8	
**w19**		10	8	
**w20**		12	8	
**w21**		14	8	
**w22**		15	9	
**w23**		15	10	
**w24**		15	12	
**w25**		15	14	
**w26-42**		15	14	
	**Food**			
		Sanders		Made by PEAT INRAE
**d1-w2**		Pullets 0/9, *ad libitum*		Starter 1N, *ad libitum*
**w3**		Pullets 0/9, rationed		Starter 1N, *ad libitum*
**w4-w6**		Pullets 0/9, rationed		Finishing 2N, *ad libitum*
**w7**		Pullets 0/9, rationed		
**w8-19**		Pullets 7/19[a], rationed		
**w20-42**		Laying hens, rationed	Roosters, rationed	
	**Nutritional supplementation**			
**w1**		Prebiotics[b] + Probiotics[c]		
**w4, w10**		Vitamin B		

n: number of birds ; d: density (number of birds/m^2) ; [a]Low energy food (2400 Kcal/Kg) ; [b]Hydrostart ; [c]Covibiote

Table S2 Prophylaxis plan in rearing

	Prophylaxis		Mode of application
**Age**	**Parental flocks**	**Broiler flocks**	
	**Disinfection of buildings**		
**w-2**	Insecticide[a]	Insecticide[a]	Sprayed
	Degreasing foam	Degreasing foam	High pressure
**w-1**	Disinfectant[b]	Disinfectant[b]	Sprayed
	**Vaccination**		
**d1**	Marek's disease	Marek's disease	Intra muscular
	Infectious Bronchitis	Infectious Bronchitis	Sprayed
**w1**	Coccidiosis	Coccidiosis	Drinking water
**w2**	Infectious Bronchitis	Infectious Bronchitis	Sprayed
**w3**	Gumboro disease	Gumboro disease	Drinking water
**w4**	Gumboro disease		Drinking water
**w5**	Rhinotracheitis		Nebulization
	Newcastle disease		Nebulization
**w9**	Infectious anemia		Drinking water
**w11**	Infectious Bronchitis		Sprayed
**w12**	Newcastle disease		Drinking water
**w14**	Encephalomyelitis		Drinking water
**w18**	Anthelmintic		Drinking water
**w20**	Newcastle disease		Intra muscular
	Infectious Bronchitis		Intra muscular
	Egg Drop Syndrome		Intra muscular
	Rhinotracheitis		Intra muscular
	Gumboro disease		Intra muscular
	**Egg disinfection**		
	Formaldehyde 15% + Potassium permanganate 50%		In formalin dispensing cabinet

w: week ; d: day ; [a]insecticide (Solfac) ; [b]Bactericidal, virucidal, fungicidal disinfectant (TH5)

Table S3 Sequenced strains description and accession numbers (sup xlsx file)

Table S4 Genetic context of ARGs detected in minor tetra-resistant STs

Origin	ST	Chr.	IncF	IncF ST	IncI1/I2	IncHI1
Parental G2	ST-69 (O17:H18)	*bla*$_{TEM-1B}$, *dfrA1*, *aadA1*, *strA*, *strB*, *sul1*, *sul2*, *mph(B)*	Ø; Ø	F64:A-:B58; F76:A-:B-	-	-
Broiler G3	ST-350	*sul2*	Ø	F64:A-:B58	-	*tet*(A), *bla*$_{TEM-1B}$
Parental G1	ST2485	-	*tet*(A), *bla*$_{TEM-1B}$, *dfrA1*, *strA*, *strB*, *sul1*	F24:A-:B58	Ø	-
Broiler G2	ST-155	*tet*(A)	*tet*(A), *bla*$_{TEM-1B}$, *dfrA14*, *aadA1*, *strA*, *strB*, *sul2*, *catA1*, *mph(B)*	F36:A27:B58	-	-
Parental G2	ST-58	-	*tet*(A), *bla*$_{TEM-1B}$, *dfrA5*, *strA*, *strB*, *sul2*	F2:A-:B58	Ø	-
Parental G3	ST-1518	-	*tet*(A), *bla*$_{TEM-1B}$, *dfrA1*, *aadA1*, *sul1*	F24:A-:B6	-	*tet*(A), *bla*$_{TEM-1B}$
Broiler G3	ST-48 (O113:H16)	-	Ø	F40:A-:B75	-	*bla*$_{CARB-2}$, *dfrA16*, *aadA1*, *aadA2*, *sul3*, *cmlA1*
Parental G3	ST-1286	*tet*(A)	-	-	*bla*$_{TEM-1B}$, *tet*(A), *dfrA1*, *aadA1*, *sul2*	-

Ø: plasmid present but not carrying any known resistance gene

Table S5 Genetic context of ARGs detected in STs with an AMP-TET resistance profile

Origin	ST	IncF	IncF ST	IncN	IncHI1	IncHI2	IncX1
Parental	ST-38	*tet*(B), bla_{TEM-1A}	F4*:A-:B80*	-	Ø	*tet*(A)[a]	-
Parental	ST-349	*tet*(B), bla_{TEM-1A}	F4*:A-:B80*	-	-	-	-
Parental G1	ST-2705	*tet*(A)	F34:A-:B-	-	bla_{TEM-1B}	Ø	-
Broiler G2	ST-1518	-	-	-	*tet*(A), bla_{TEM-1B}	-	-
Parental G2	ST-1636	Ø[b]	/[b]	*tet*(A)	bla_{TEM-1B}	-	-
Parental G1	ST-48 (O61:H11)	*tet*(B); Ø	F4:A-:B75*; F82*:A-:B-	-	-	-	bla_{TEM-1D}
Parental G1	ND- (H48)	*tet*(B)	F82*:A-:B-	*tet*(A)	bla_{TEM-1B}	-	-
Broiler G3	ST-10	Ø[a,b]	/[b]	-	*tet*(A), bla_{TEM-1B}	-	-

*: closest allele but not identical
Ø: plasmid present but not carrying any known resistance gene
a: plasmid not found in all isolates
b: conjugative system and replication genes not related to other IncF plasmids

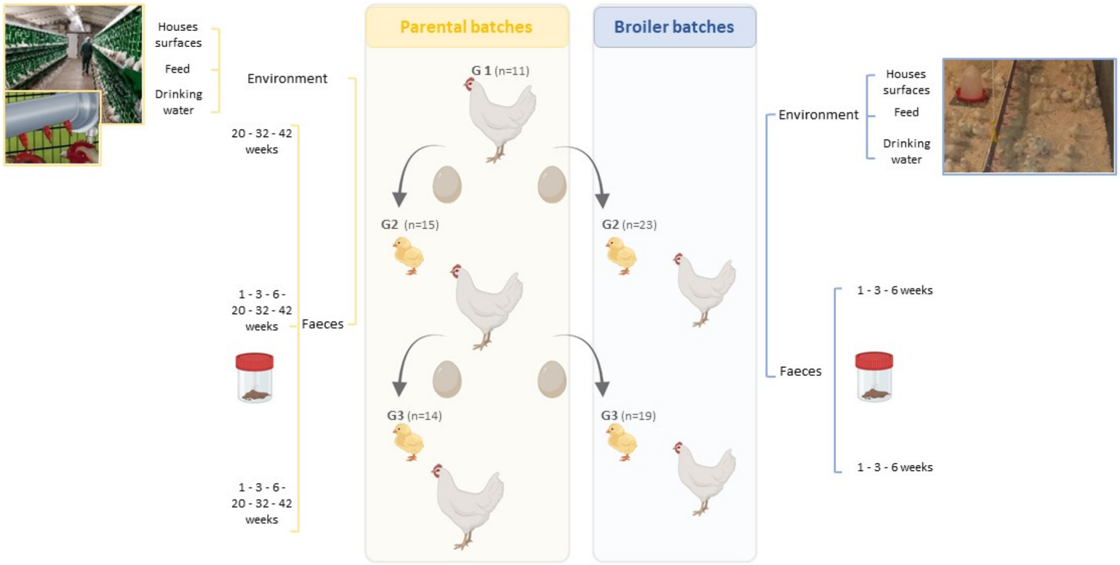

Figure S1 Sampling plan in the experimental facility. Chickens were followed over three generations including two sibling batches, one for reproduction which are reared in protected houses during 42 weeks (Parental batches) and one for broiler production reared in standard conditions during 6 weeks (Broiler batches). Individual chicken fecal samples were collected at different weeks of age, first on 11 pedigree broiler breeder females (Generation 1) at 20, 32 and 42 weeks and then on their progeny reared in the 2 conditions at 1, 3 and 6 weeks. This was repeated for the next generations (G2 and G3). Eggs collected for analysis were extra eggs of the two batches of G2 and G3. The environmental samples corresponding to buildings surfaces and drinking water were taken before animal's entry.

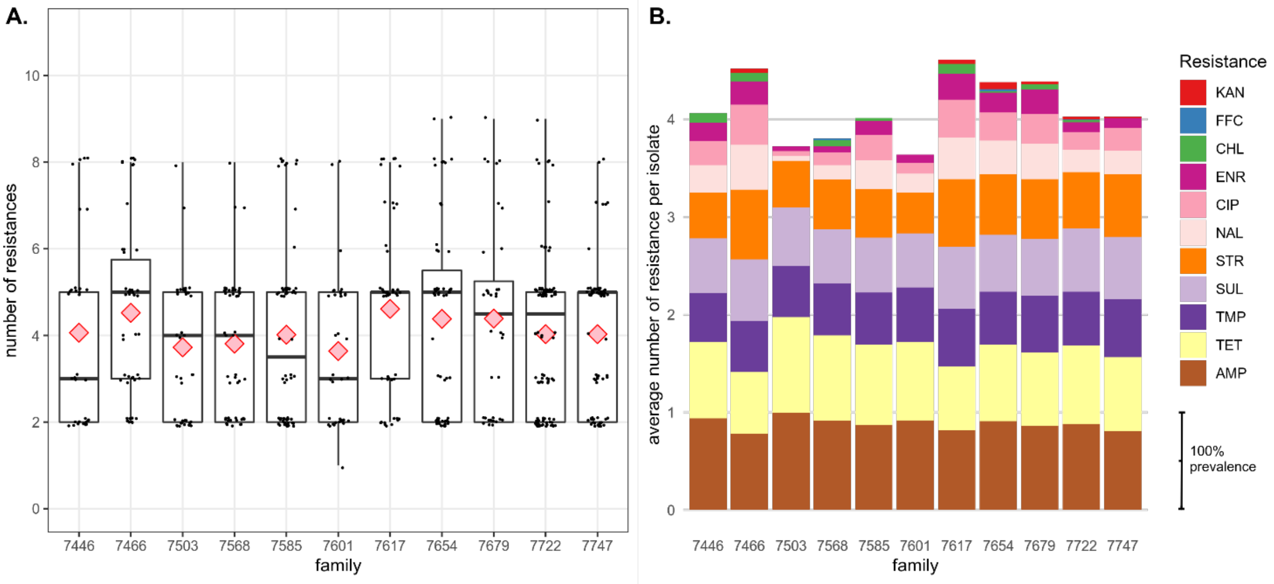

Figure S2 Number **(A)** and distribution **(B)** of resistances among *E. coli* isolates per chicken family. Median and average resistance number is represented by a thick horizontal bar and a pink diamond in (A), respectively. Bars in (B) represent the cumulative prevalence of isolates from each family resistant to at least two antibiotics, calculated antibiotic by antibiotic.

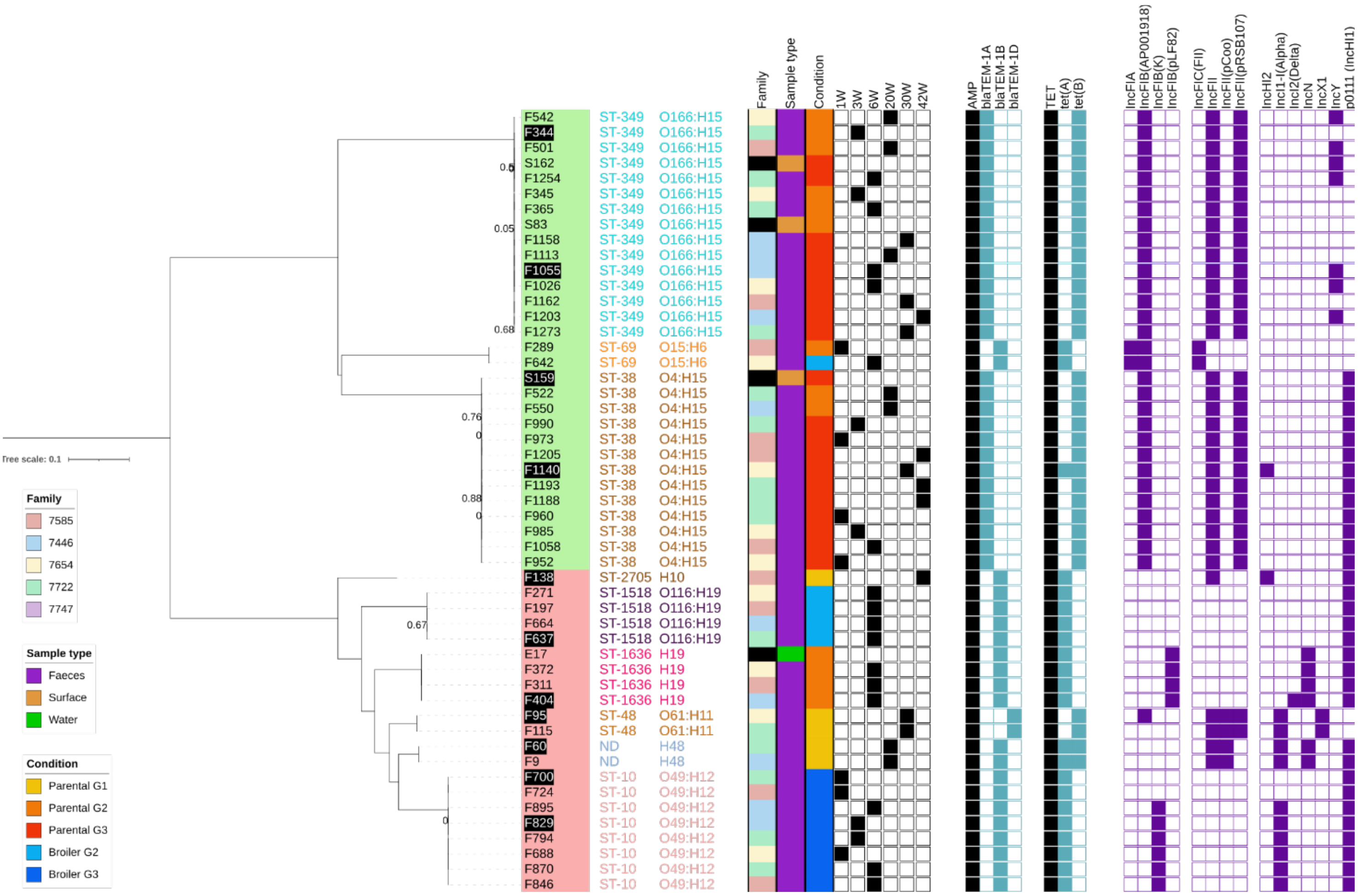

Figure S3 Phylogenetic relationship of 51 *E. coli* isolates resistant to AMP and TET, originating from faeces and from drinking water or breeding surfaces before animal's entry. The tree was constructed with a maximum likelihood algorithm from the core genome SNP alignment. *E. fergusonii* was used as an outgroup but is not shown on the figure to improve branch length visibility. Branch confidence values of 0.9 or higher are not displayed. For each antibiotic, the resistance phenotype is symbolized with a black filled square while presence of associated resistance gene(s) is indicated with blue filled squares. Samples highlighted in black were assembled using both short and long reads.

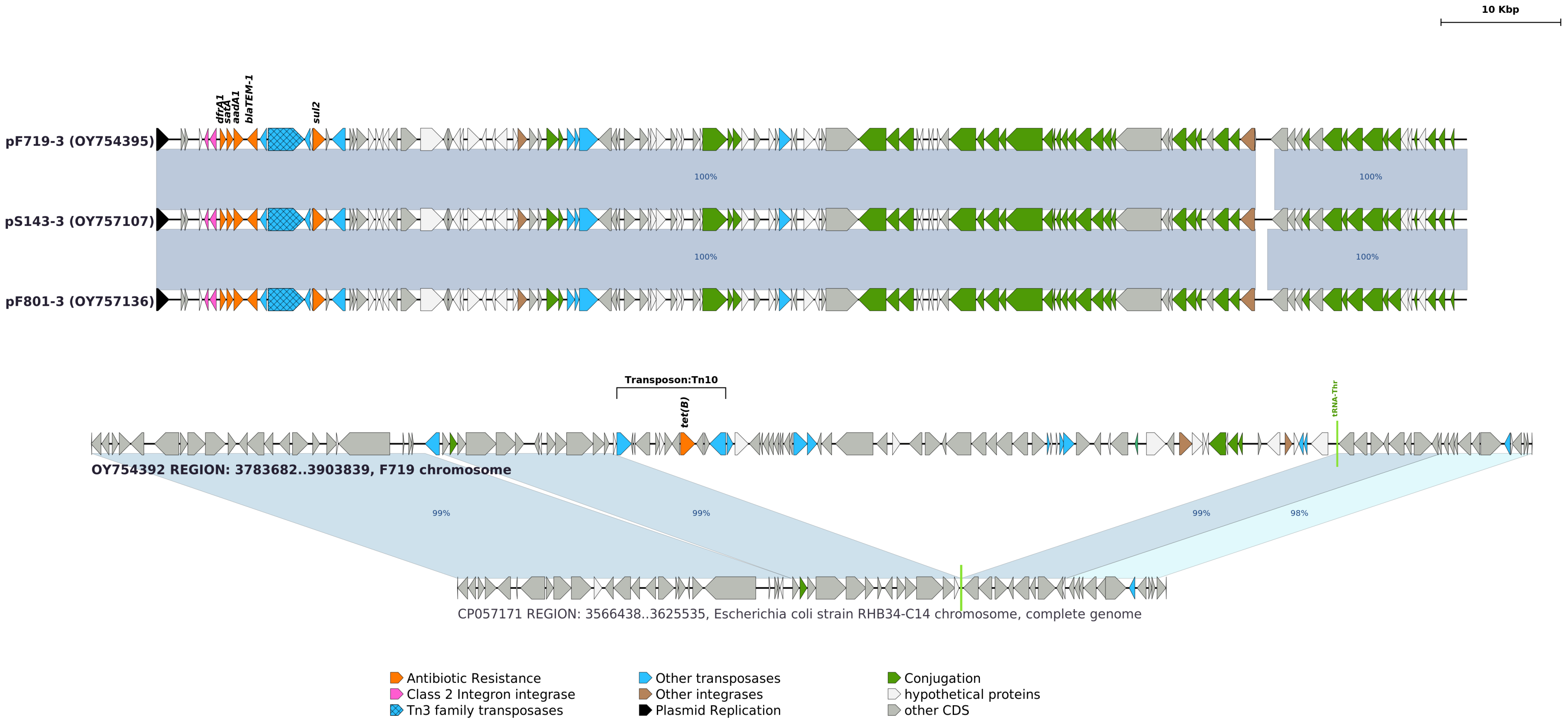

Figure S4 ARG-carrying plasmid and IME structures in ST-453 isolates.

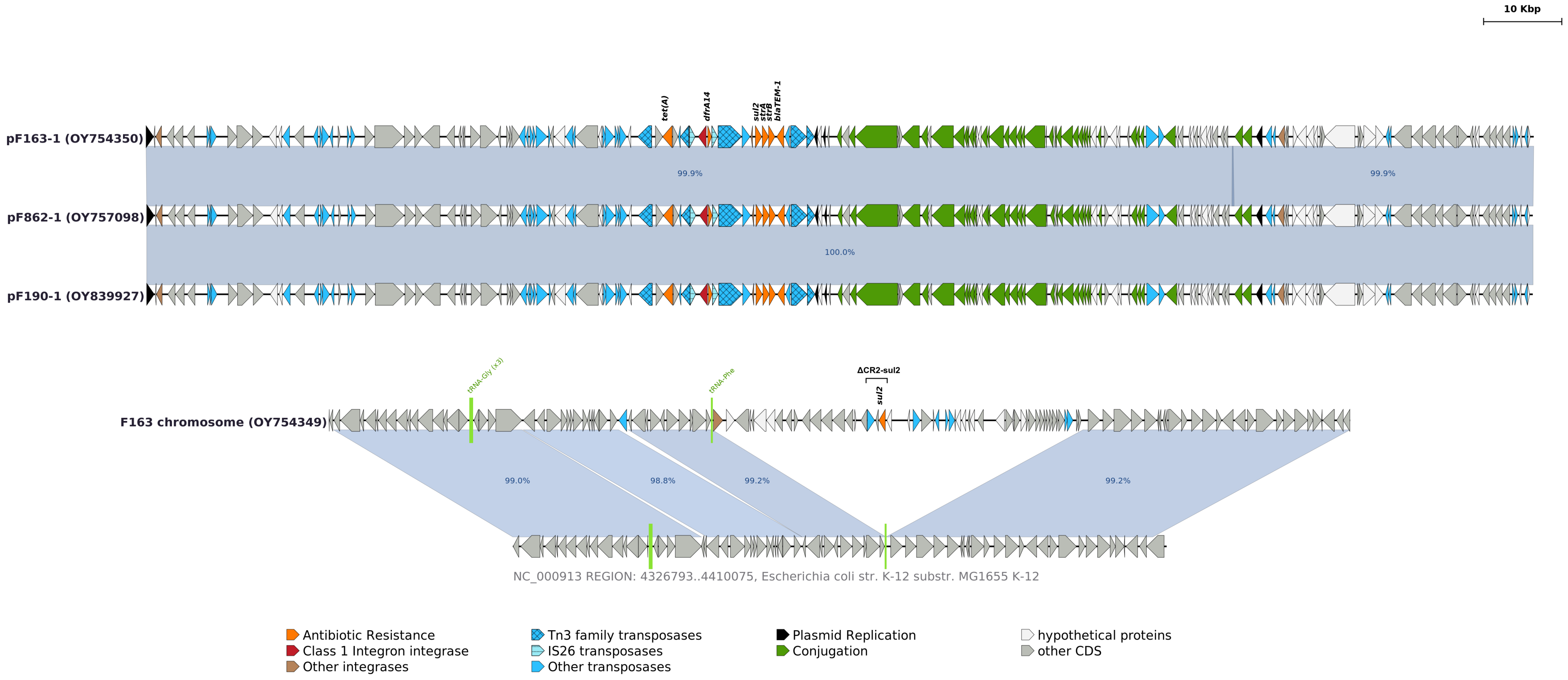

Figure S5 ARG-carrying plasmid and IME structures in ST-1611 isolates.

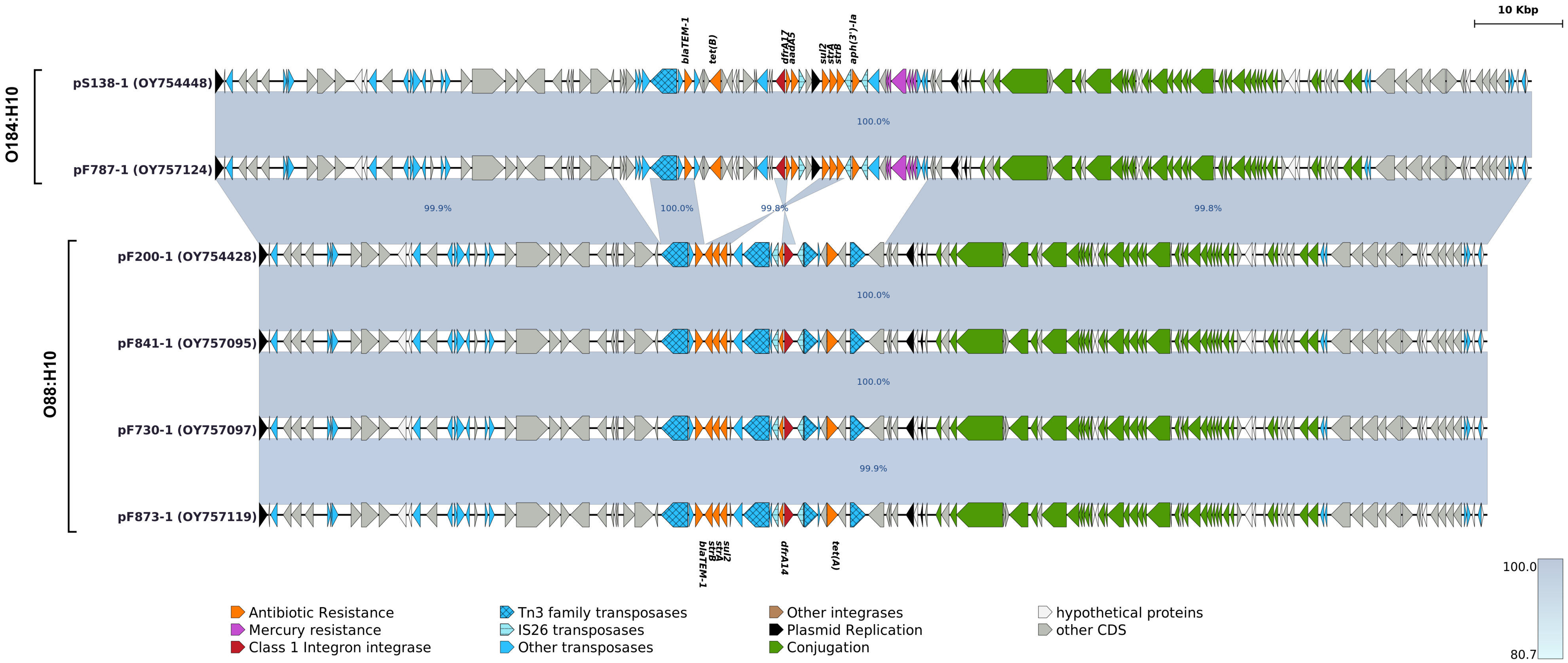

Figure S6 ARG-carrying plasmid structures in ST-162 isolates.

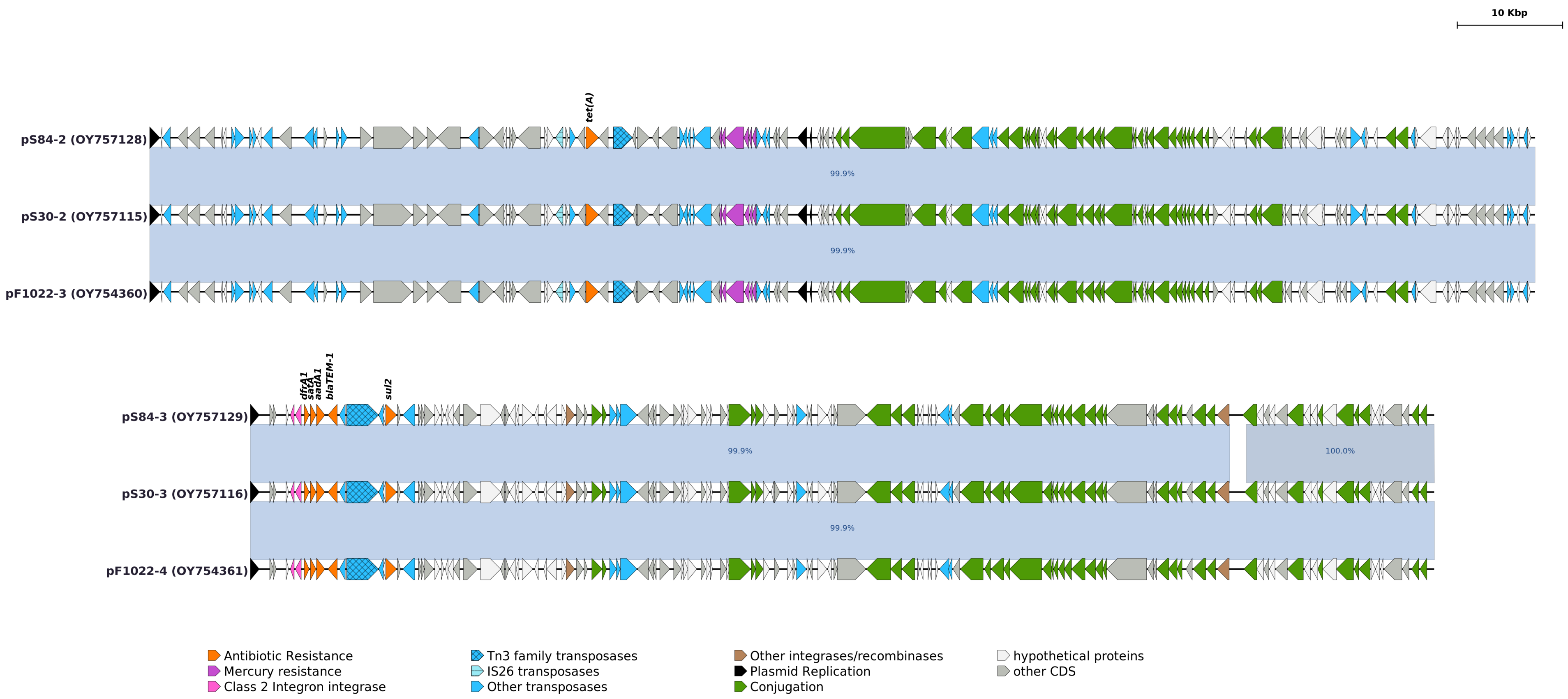

Figure S7 ARG-carrying plasmid structures in ST-93 isolates.

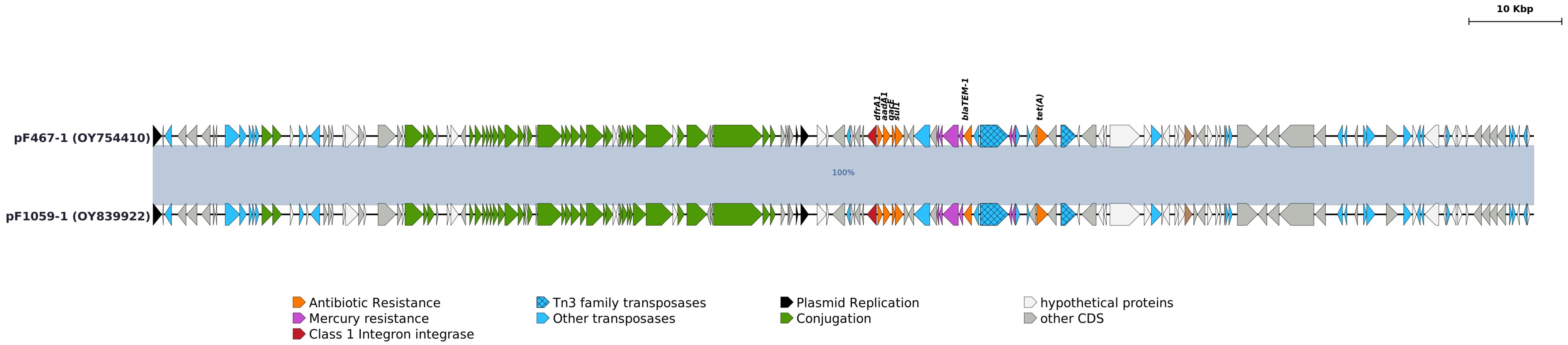

Figure S8 ARG-carrying plasmid structures in ST-206 isolates.

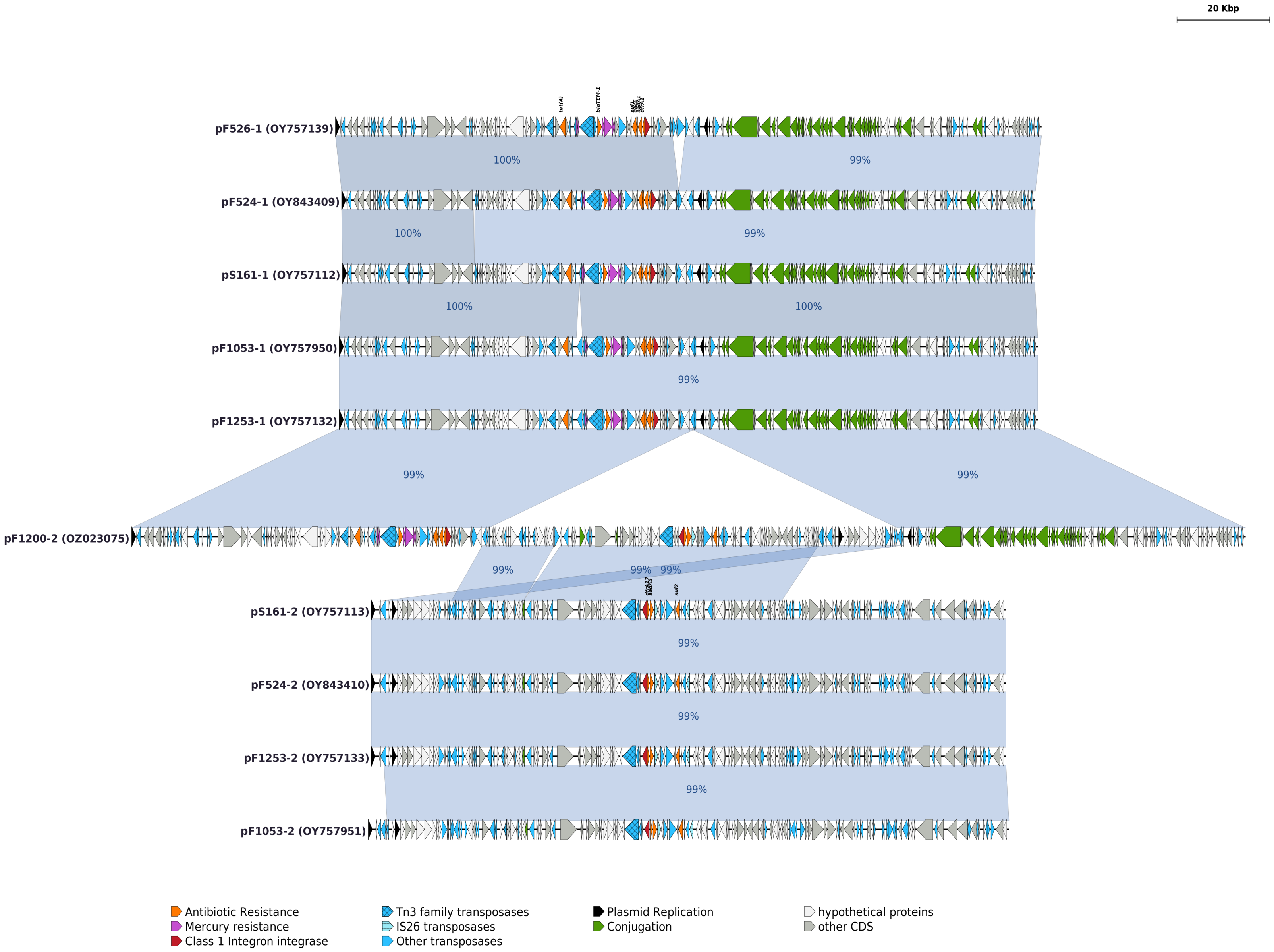

Figure S9 ARG-carrying plasmid structures in ST-2701 isolates.
